# Proinflammatory Cytokines Predict Brain Metabolite Concentrations in the Anterior Cingulate Cortex of Patients With Bipolar Disorder

**DOI:** 10.3389/fpsyt.2020.590095

**Published:** 2020-12-08

**Authors:** Sara Poletti, Mario Gennaro Mazza, Benedetta Vai, Cristina Lorenzi, Cristina Colombo, Francesco Benedetti

**Affiliations:** ^1^Psychiatry & Clinical Psychobiology, Division of Neuroscience, IRCCS San Raffaele Scientific Institute, Milan, Italy; ^2^Vita-Salute San Raffaele University, Milan, Italy

**Keywords:** spectroscopy, *myo*-inositol, glutamate, inflammation, mood disorder

## Abstract

Bipolar disorder (BD) is a severe psychiatric illness characterized by abnormalities in the immune/inflammatory function and in brain metabolism. Evidences suggest that inflammation may affect the levels of brain metabolites as measured by single-proton magnetic resonance spectroscopy (^1^H-MRS). The aim of the study was to investigate whether a wide panel of inflammatory markers (i.e., cytokines, chemokines, and growth factors) can predict brain metabolite concentrations of glutamate, *myo*-inositol, *N*-acetylaspartate, and glutathione in a sample of 63 bipolar patients and 49 healthy controls. Three cytokines influenced brain metabolite concentrations: IL-9 positively predicts glutamate, IL-1β positively predicts *Myo*-inositol, and CCL5 positively predicts *N*-acetylaspartate concentrations. Furthermore, patients showed higher concentrations of glutamate, *Myo*-inositol, and glutathione and lower concentrations of *N*-acetylaspartate in respect to healthy controls. Our results confirm that inflammation in BD alters brain metabolism, through mechanisms possibly including the production of reactive oxygen species and glia activation.

## Introduction

Bipolar disorder (BD) is a severe psychiatric illness characterized by abnormalities in the immune/inflammatory function and in brain metabolism. Increased inflammatory markers have been found in the cerebrospinal fluid (CSF) and peripheral blood of BD patients irrespective of mood (i.e., in depression, mania, and euthymia) (1, 2). Peripheral cytokines can enter the brain by volume diffusion or via active cytokine transporters at the blood–brain barrier (3), and high peripheral levels of cytokines are associated with a lack of response to antidepressant treatment (4–6), with brain abnormalities affecting both white matter (WM) and gray matter (GM) (7, 8), and with the cognitive impairment associated with brain abnormalities (9), thus suggesting that peripheral biomarkers can be exploited to study the low-grade inflammation that associates with the most detrimental effects of BD on brain structure and function (10, 11).

Altered concentrations of brain metabolites have been widely reported in BD both post mortem and *in vivo* using single-proton magnetic resonance spectroscopy (^1^H-MRS), a non-invasive brain imaging technique that can detect alterations in brain biochemistry in the presence of apparently normal anatomy. Resonances in the ^1^H-MR spectrum can be reliably quantified for several metabolites with brain concentrations in the millimolar range in specific brain regions including *N*-acetylaspartate (NAA), creatine (Cr), glutamine (Gln), glutamate (Glu), Glx (i.e., Gln + Glu), glutathione (GSH), *Myo*-inositol (mI), and creatine + phosphocreatine, a measure of energy utilization that has historically been used as an ^1^H-MRS internal standard. The volume of interest (VOI) most investigated by ^1^H-MRS studies in BD is the anterior cingulate cortex (ACC) (12, 13), because of its link to mood regulation (14) and antidepressant response in BD (15).

One of the most reported findings of brain metabolite abnormalities in BD is an increased level of glutamate, the principal excitatory neurotransmitter in the central nervous system. Most studies on BD have reported increased Glu and Glx in multiple brain voxels during both mood episodes (mania and depression) and euthymia (12, 13, 16, 17), in pediatric BD (18) as well as in depressed drug-free BD [Glx; (19)]. Alterations in the glutamatergic system have been suggested to contribute to the pathophysiology of depression (20) as suggested by findings showing that inhibition of glutamate release and reduction in glutamate levels in depressed BD patients parallel the antidepressant response (21–23).

Other brain metabolites previously investigated in BD include mI, NAA, and GSH. Inositol is located within astrocytes and participates in glial osmoregulatory functioning, phospholipid metabolism, and signal transduction (24) and is usually interpreted as a glial specific marker. Contrasting results have been reported regarding mI alterations in BD [for a review, see (25, 26)]. Increased mI concentrations have been reported in euthymic and manic bipolar children, no difference has been reported in adult bipolar patients compared with controls (27), but reduced concentrations in patients taking lithium (28) and irrespective of lithium treatment (29) have been reported.

GSH is the main antioxidant in the brain produced by neurons, and microglia require astrocytic support providing the precursor amino acids (30, 31) and play an important role in the maintenance of oxidative balance. If the equilibrium between reactive oxygen species (ROS) and antioxidants is perturbed, oxidative stress may occur, resulting in toxicity and cell damage (32). GSH is also involved in the prevention of mitochondrial damage and in the protection against glutamate-induced excitotoxicity (33, 34). Few studies have investigated GSH in BD patients showing either unchanged or increased levels as compared with controls [for a meta-analysis, see (35)] in the ACC.

Finally, NAA is an abundant neuronal metabolite considered as a marker of mitochondrial activity and neuronal integrity (36). Accordingly, decreased levels of NAA have been suggested to reflect impaired mitochondrial energy production (36, 37). Decreased NAA levels have been reported in several regions throughout the brain (38). Less consistent data have been reported in the ACC, where both reduced levels of NAA/Cr (39) in adolescents and increased NAA levels in depressed (16) and euthymic adults (28) have been shown.

Alterations in brain metabolism could be influenced by the biological effects of inflammation mediating a detrimental role on mood disorders (40, 41). Accordingly, in patients with hepatitis C and comorbid depression, interferon (IFN)-alpha administration was associated with significant increases in Glu/Cr in basal ganglia and ACC (42). Similarly, in patients affected by major depression, levels of C-reactive protein are associated with Glu levels in the basal ganglia (43). In BD, a positive association was observed between microglial activation and NAA + *N*-acetyl-aspartyl-glutamate (NAAG) (44), whereas NAA decreases prostaglandin E2, COX-2, intracellular calcium, and NF-jB in stimulated human astroglial cells (45), suggesting that NAA could play a role in the modulation of inflammation within the central nervous system. Also, GSH is involved in the inflammatory process. Indeed, on the one hand, GSH levels increase following inflammation to counteract oxidative stress (46), while on the other hand, GSH stimulates energy metabolism in T cells (47). Finally, elevated levels of mI seem to suggest the presence of glial activation, which is commonly associated with neuroinflammation (48).

Despite these promising findings, to our knowledge, no study so far investigated the association between a wide panel of peripheral inflammatory markers and brain metabolites in BD. The aim of this study was then to investigate whether plasma levels of cytokines and chemokines are associated with ACC concentrations of Glu, NAA, mI, and GSH in a sample of BD patients.

## Materials and Methods

### Participants and Data Collection

The sample was composed of 63 consecutively admitted (age 18–65) inpatients with BD (*Diagnostic and Statistical Manual of Mental Disorders*, 5th edition [DSM-5] criteria) and 49 healthy controls (HCs). Fifty-one patients were in a depressive state, four were euthymic, and eight were in a manic phase. Thirty-two patients were taking lithium from at least 6 months, 37 were taking antidepressants, 11 were taking atypical antipsychotics, and 31 were taking antiepileptic drugs. The recruitment took place from January 2015 to December 2018. Exclusion criteria were current diagnosis of any additional psychiatric disorders including alcohol and/or substance dependence or abuse in the last 6 months, intellectual disability, pregnancy, major medical and neurological disorders, and medical conditions affecting the immune system. All participants underwent MR scanning, while only patients underwent blood sampling. Fasting blood samples were taken in the morning (between 7:00 and 9:00 a.m.). After a complete description of the study, written informed consent was obtained. All research activities have been approved by the Ethical Committee.

### Laboratory Determinants

Plasma concentrations of the following immune analytes were determined using the bead-based Luminex system based on xMAP technology (Bio-Rad Laboratory, Hercules, CA, USA). Cytokines: interleukin (IL)-1β, IL-1rα, IL-2, IL-4, IL-5, IL-6, IL-7, IL-8, IL-9, IL-10, IL-12, IL-13, IL-15, IL-17, IFNγ, tumor necrosis factor (TNF)-α. Chemokines: C-C motif ligand (CCL)2, CCL3, CCL4, CCL5, and CCL11 and C-X-C motif ligand (CXCL) 10. Growth factors: basic fibroblast growth factor (bFGF), granulocyte colony-stimulating factor (G-CSF), granulocyte-macrophage colony-stimulating factor (GM-CSF), platelet-derived growth factor beta (PDGF-β), and vascular endothelial growth factor (VEGF).

Assays were performed on Luminex 200 system. Samples were analyzed according to manufacturer's instructions. The intra-assay coefficient of variation was 2.3–4.8%, and inter-assay coefficient of variation was 4.9–28.2%.

### Magnetic Resonance Spectroscopy Procedure

MRS was performed at the C.E.R.M.A.C. (Centro d'Eccellenza di RisonanzaMagnetica ad Alto Campo) in San Raffaele Hospital in Milan on a 3.0 Tesla magnet (Ingenia, Philips) with a standard quadrature head coil. A structural MRI study was performed first to rule out brain lesions and to localize the VOI for the spectroscopy study, acquiring sagittal T1 images, axial T2 fast spin-echo (FSE) images parallel to the bicomessural line, and coronal fluid-attenuated inversion recovery (FLAIR) images orthogonal to the axial ones.

**Figure 1:**
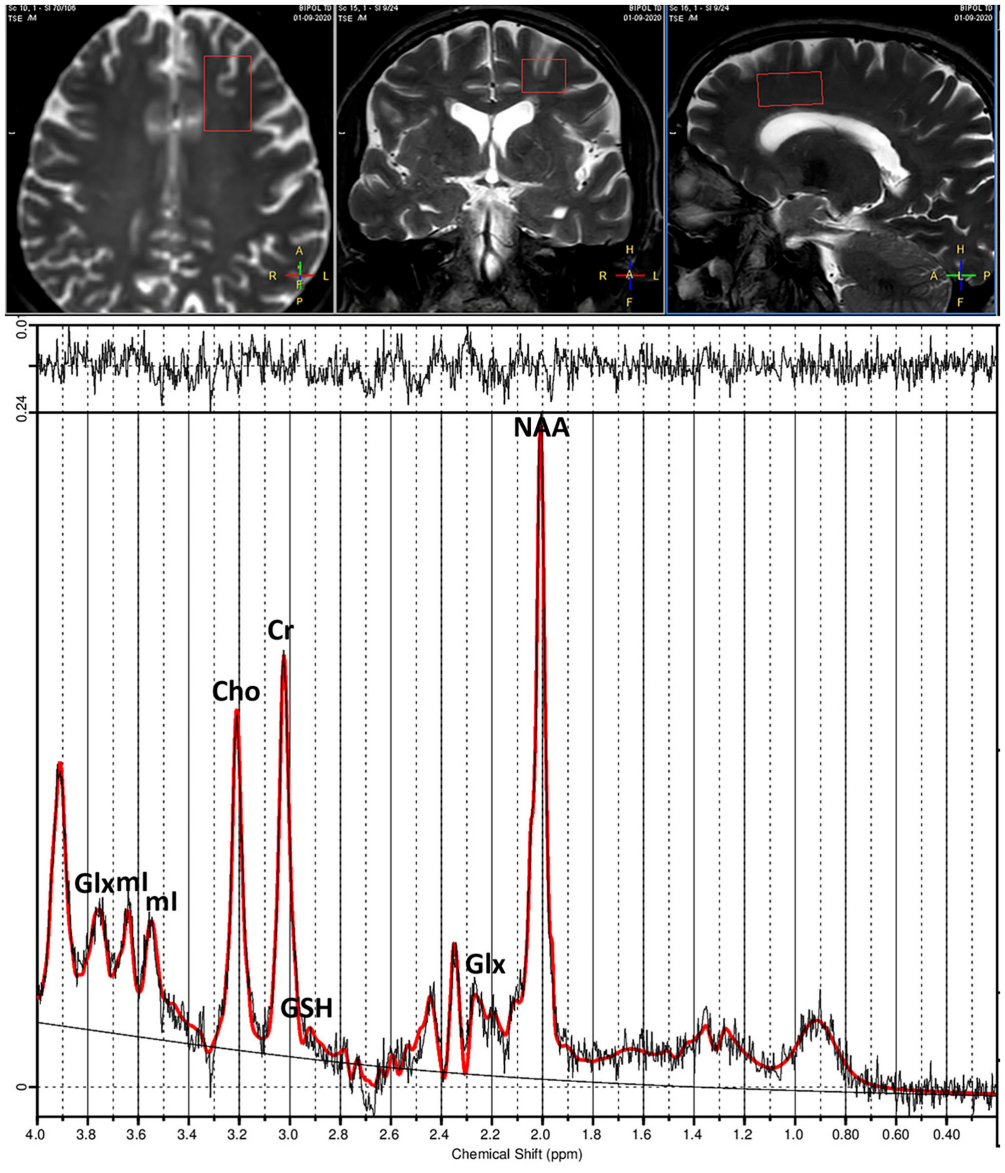
**(Top)** T1-weighted images show placement of voxel in anterior cingulate cortex (ACC). **(Bottom)** Example of proton spectra from ACC and peak intensities of brain metabolites. Glx, Glutamate+Glutamine; mI, myo-inositol; Cho, choline; Cr, creatine; GSH, glutathione; NAA, N-acetylaspartate.

^1^H-MRS data were acquired using a point resolved spectroscopy (PRESS) sequence (repetition time [TR] 2,000 ms, echo time [TE] 42 ms, 128 acquisitions) from VOI of 30 × 20 × 15-mm size positioned at the level of the ACC (Figure 1). Unsuppressed water reference spectra were acquired from the same VOI.

#### ^1^H-MRS Quantification

^1^H-MR spectra were then analyzed using LCModel version 6.3.0 (49) and a basis set including alanine, aspartate, Cre, phosphocreatine, glucose, Gln, Glu, glycerophosphocholine, phosphocholine, mI, lactate, NAA, NAAG, scyllo, taurine, macromolecules, and lipid signals. The LCModel method analyzes an *in vivo* spectrum as a linear combination of model *in vitro* spectra from individual metabolite solutions. Complete model spectra, rather than individual resonances, are used in order to incorporate maximum prior information into the analysis. A nearly model-free constrained regularization method automatically accounts for the baseline and lineshape *in vivo* without imposing a restrictive parameterized form on them. LCModel is automatic (non-interactive) with no subjective input. Approximately maximum-likelihood estimates of the metabolite concentrations and their uncertainties (Cramér-Rao lower bounds) are obtained. The unsuppressed water signal measured from the same VOI was used as an internal reference for the quantification (assuming 80% brain water content). The LCModel analysis calculates the best fit to the experimental spectrum as a linear combination of model spectra (solution spectra of brain metabolites). The final analysis is performed in the frequency domain; however, raw data (FIDs) are used as standard data input. Voxel position and metabolites peaks are presented in Figure 1.

Tissue segmentation was performed in order to estimate the proportion of GM, WM, and CSF in the voxel. Brain tissue in the three-dimensional T1-weighted brain images was segmented using the Gannet Co Register and Gannet Segment functions in the Gannet 2.0 toolbox (50) in SPM12. The CSF brain tissue fraction was calculated for each voxel [fCSF = %CSF/(%GM + %WM + %CSF)]. Concentrations were then corrected for CSF fraction with the following formula:

Corrected concentration = metabolite concentration ^*^ (1/[1 – fCSF])

To ensure the accuracy of the measurements obtained, only metabolite results with values of Cramér–Rao lower bound <20% were considered.

### Statistical Analyses

Cytokines having non-detected values >20% have been excluded similarly to previous studies (51).

Univariate statistical analyses were performed using STATISTICA (StatSoft Statistica 11, Tulsa, OK, USA). Group differences for brain metabolites were performed through *t*-tests and ANCOVAs to account for the effect of possible confounding factors (age and gender). Pearson's r was used to investigate the association between brain metabolites and clinical-demographic variables. Tests were two-tailed, and an alpha level of 0.05 indicated statistically significant results. In order to control for multiple comparisons, *p*-values were corrected through false discovery rate procedure (52).

The predictive effect of baseline peripheral measures of inflammation on brain metabolite concentrations was tested in three steps.

First, through a regression analysis, we investigated the effect of two cytokines (i.e., IL-9 and TNF-α) previously associated with BD (53) with glutamate concentrations.

Second, to explore the effect of each cytokine in affecting mI, NAA, and GSH brain concentrations, we entered all analytes as predictors in an elastic net penalized regression (54). Before performing the analyses, all the data were normalized (i.e., min–max normalization). In elastic net models, the applied regularizations (L1 and L2) force shrinkage of the coefficients, which can also be estimated to zero, reducing overfitting and eliminating irrelevant or redundant variables. This not only increases the model interpretability but also allows dealing with multicollinearity induced by highly correlated variables (i.e., cytokines and chemokines). We implemented a non-parametric bootstrap procedure (5,000 resamples with replacement) in order to provide estimates of mean log odds ratio, related 95% confidence intervals, and variable inclusion probability (VIP) (54). In regularized regression, asymptotically valid *p*-values are not available; VIP allows providing a measure of stability of coefficient as an estimate of posterior probability of the predictors included in the model (55). We applied a threshold of 75% to VIP (55). Age, sex, onset, number of episodes, body mass index (BMI), and lithium plasma levels were entered as nuisance covariates. At each bootstrap, λ values used in elastic net model were defined through a 5-fold nested cross-validation procedure with the better model fitting defined as minimum expected deviance, as calculated by cross-validations. Minimizing the λ deviance is equivalent to maximizing the λ log-likelihood. The algorithm function was solved through the coordinate descent algorithm as implemented in MATLAB (56).

Third, we then confirmed the relevance of these associations and assessed the direction of effects by modeling the effect of cytokines, surviving the VIP threshold on brain metabolite concentrations in a regression analysis within the context of the generalized linear model. All nuisance covariates were entered in the analysis.

## Results

Clinical and demographic characteristics of the sample are resumed in Table 1. The HC group was younger than the patients (*t* = 6.19, *p* <0.001) and with a lower number of females (χ = 10.43, *p* = 0.001). We excluded from the analysis IL-10, IL-15, GM-CSF, and VEGF showing non-detected values >20%. No differences between depressed, manic, and euthymic patients were observed in brain metabolite concentrations nor in immune/inflammatory marker levels. Controlling for age and gender, BD patients and HCs had similar GM (*p* = 0.11), WM (*p* = 0.088), and CSF (*p* = 0.22) content within the analyzed voxel.

**Table 1:** Clinical and demographic characteristics of the sample and results of proton magnetic resonance spectroscopy in the anterior cingulate cortex divided according to diagnosis.

	**BD (*N* = 63)**	**HC (*N* = 49)**		
	**Mean ± SD**	**Mean ± SD**	***t*/*χ p***	***q***
Age	46.81 ± 11.94	33.7 ± 11.14	6.19 <0.001	<0.001
Sex	M = 17 F = 46	M = 28 F = 21	10.43 0.001	<0.001
Onset (years)	29.01 ± 11.15	-	-	
Duration of illness (years)	17.80 ± 11.93	-	-	
N. depressive episodes	6.24 ± 5.64	-	-	
N. manic episodes	3.54 ± 4.29	-	-	
BMI	26.25 ± 5.34	-	-	
HDRS	16.86 ± 9.24	-	-	
Gray matter	21.06 ± 7.31	18.35 ± 9.31	1.70 0.090	NS
White matter	77.45 ± 7.95	81.02 ± 10.28	2.05 0.043	NS
Cerebrospinal fluid	1.44 ± 1.51	0.75 ± 1.06	2.67 0.008	0.005
Glutamate	8.11 ± 0.99	7.68 ± 1.04	2.21 0.029	0.029
Glx	9.90 ± 1.31	9.20 ± 1.17	2.95 0.003	0.004
NAA	6.62 ± 0.43	7.14 ± 0.54	5.67 <0.001	<0.001
GSH	1.70 ± 0.26	1.54 ± 0.16	3.77 <0.001	<0.001
*Myo*-inositol	5.24 ± 1.06	4.29 ± 0.86	5.13 <0.001	<0.001

*Mean values presented are not values adjusted by age, gender, and gray matter brain tissue fraction. BD, bipolar disorder; HC, healthy control; HDRS, Hamilton Depression Rating; Glx, Gln + Glu; NAA, N-acetylaspartate; GSH, glutathione*.

No patient was excluded because of poorly fitting metabolites peaks. Full width at half maximum was 0.042 ± 0.007 parts per million (range, 0.031–0.061) for the BD group and 0.038 ± 0.006 parts per million (range, 0.031–0.055) for the HC group. The signal-to-noise ratio was 19.6 ± 2.9 parts per million (range, 13–29) for the BD group and 22.7 ± 2.9 parts per million (range, 15–28) for the HC group. BD patients had higher linewidth (*t* = 2.5, *p* = 0.013) and lower signal-to-noise ratio (*t* = 5.55, *p* < 0.001) than had HCs.

Accounting for age and gender patients showed higher concentrations of Glu (*F* = 5.85, p__FDR__ = 0.021), Glx (*F* = 8.39, p__FDR__ = 0.010), GSH (*F* = 6.96, p__FDR__ = 0.015), and mI (*F* = 4.93, p__FDR__ = 0.028) and lower concentrations of NAA (*F* = 8.48, *p* = 0.010). No association between brain metabolite concentration and plasma levels of lithium was observed. An older age was associated with higher concentrations of mI (*r* = 0.48, p__FDR__ < 0.001). A higher number of manic episodes were associated with higher concentrations of GSH (*r* = 0.38, p__FDR__ = 0.03).

At multiple regression, Glu concentrations were predicted by levels of IL-9 (*b* = 0.001, SE = 0.0007, Wald = 5.03, *p* = 0.025), whereas no effect of TNF-α was observed.

Results of elastic net penalized regression (Supplementary Tables 1–3) showed that five cytokines survived the VIP threshold of 75%: IL-1β, IL-4, and bFGF predicted mI; TNF-α and CCL4 predicted GSH; and CCL5 predicted NAA concentrations. A significant association between IL1-β and mI concentrations (*b* = 1.94, SE = 0.77, Wald = 6.21, *p* = 0.013) and between CCL5 and NAA (*b* = −0.000014, Wald = 4.58, *p* = 0.032) was also confirmed by the regression analysis.

## Discussion

The main finding of the present study is the presence of an association between peripheral inflammatory markers and brain metabolites in BD. Here, we showed that higher IL-9, IL-1β, and CCL5, respectively predict higher Glu, mI, and NAA concentrations in the ACC. Also, we observed higher levels of Glu, Glx, mI, and GSH and lower levels of NAA in BD patients compared with HCs. Given that Glu and mI are primary targets for the treatment of the disorder (57, 58), these findings deepen our understanding of the relationship between low-grade inflammation and brain metabolism in BD and are in agreement with existing literature about brain homeostatic processes.

Immune disturbances, especially cytokine alterations (1, 59), have been widely reported in BD and have been associated with symptom severity (60) and mood episodes (61). Although not previously investigated in BD, we recently showed (53) that IL-9 is associated with the likelihood of having BD. IL-9 is a pleiotropic cytokine produced mainly by Th2 cells, but also by Th17, T regulatory cells, and Th9 (62). The IL-9 receptor is expressed in several cell types including astrocytes where the binding of T cell-derived IL-9 leads to Th17 cell migration *in vitro* (63). In turn, IL-17 released by TH17 may reduce the expressions of glutamate transporters (64), thus possibly explaining the association we observed between higher levels of IL-9 and brain concentrations of Glu.

Among the cytokines that have been involved in BD, IL-1β is a proinflammatory cytokine released by activated macrophages and microglia, which plays a key role in the onset of inflammatory processes, activates the production of TNF-α, and triggers astrocyte activation (65). Although in the CNS we may not observe all the manifestations of peripheral inflammation (i.e., no swelling), morphological changes in microglia, together with increased expression of IL-1β, is frequently observed in both human and animal models of brain diseases (66, 67). When glia is activated, its volume increases; glial cell volumes are maintained by mI; hence, activated glia with enlarged cell volumes may show elevated mI levels. Inflammation might then affect brain metabolism through a change in glia function, as also suggested by a recent study showing that stimulation with IL-1β and TNF-α produces a reactive astrocyte phenotype associated with both the release of inflammatory mediators and neurosupportive characteristics toward axonal growth and neuronal viability and functionality (68).

In the last years, chemokines have been shown to play a central role in BD (69). However, the effects of chemokines in the brain are complex, as these proteins have both neuroprotective and neurotoxic properties. Here, we showed a positive association between CCL5, a chemokine involved in the recruitment of T cells and NAA brain concentrations. NAA is employed as a marker for neuronal health, and a large number of RANTES-responsive genes in cultured neurons have been suggested to be involved in neuronal differentiation and survival (70). Furthermore, CCL5 protects neurons and astrocytes from NMDA-induced apoptosis (71), and treatment of primary cortical neuronal cultures with CCL5 enhances neuronal survival and reduces neuronal cell death (72). In agreement with a neuroprotective role for this chemokine, CCL5 levels may promote neuronal health as suggested by its positive association with NAA concentrations.

In agreement with the literature (see Introduction), Glu, Glx, GSH, and mI are higher in BD patients than controls, whereas NAA is lower. Glu and GSH are linked by the system Xc- transporter. Present in microglia and astrocytes, the Xc- transporter mediates the absorption of cysteine, which is needed for GSH production, extruding Glu (73, 74). During immune activation, microglia and macrophages express the excitatory amino acid transporters (EAATs), glutamine synthetase, and system Xc- transporters. Indeed, the release of inflammatory cytokines following immune activation precipitates oxidative stress with the production of ROS (75–77). Cells exposed to chronic inflammation would then require more GSH to buffer rising ROS and prevent cellular damage. An accelerated rate of GSH synthesis, in turn, would lead to a greater release of glutamate into the synapse through the system Xc transporter in exchange for cysteine. ROS, however, have been suggested to inhibit glutamate uptake in astrocytes (78), thus leading to increased glutamate levels. Furthermore, extracellular Glu increases GSH synthesis in a dose-dependent manner in macrophages that co-express both EAATs and Xc- antiporter (79) possibly through a cooperative mechanism between EAATs and Xc- system. EAATs, through the inward transport of Glu, would directly provide intracellular Glu for GSH synthesis.

The parallel increase of mI, Glu, Glx, and GSH levels in patients with BD may suggest the presence of an altered astrocytic function in response to an inflammatory process with a consequent alteration in glutamatergic neurotransmission. Furthermore, a decrease NAA concentration may reflect neuronal dysfunction, injury, or loss following the release of neurotoxic inflammatory mediators released by activated glia.

The major limitation of the study is a significant difference in age and sex between patients and controls, which has been taken into account, adding age and sex as nuisance covariates in the analyses. Also, differences in linewidth and signal-to-noise ratio between patients and controls could have influenced the results. The lack of information regarding previous psychopharmacological treatments received by the patients could have partially influenced the results, as recent studies showed that highly complex medication regimens are often required during naturalistic outpatient treatment of BD depression (80). However, in the present study, no association was observed between cytokines or brain metabolites and pharmacologic treatment. Brain metabolites may change during the depressive or the manic episodes; however, in the present study, only a small group of manic and euthymic patients was present; therefore, it was not possible to investigate phase difference. Future studies are needed to clarify this issue. Limitations also include issues such as generalizability, possible undetected past comorbidities, and population stratification. Finally, in the present study, we investigated brain metabolite concentrations in only one voxel, thus hampering the generalizability of the results to the whole brain. Further studies are needed to better understand whether the association between peripheral inflammatory markers and brain metabolites that we observed here is present also in other regions and whether differences between brain regions exist. Overall, our findings strengthen the impact of biomarker research into clinical practice and provide new insights for the development of innovative therapeutic strategies for bipolar disorder.

## Data Availability Statement

The raw data supporting the conclusions of this article will be made available by the authors, without undue reservation.

## Ethics Statement

The studies involving human participants were reviewed and approved by Comitato Etico Ospedale San Raffaele OSR. The patients/participants provided their written informed consent to participate in this study.

## Author Contributions

SP and FB designed the study. FB obtained the funding. CC was responsible for patient selection and collection of clinical data. CL performed all biological analyses. SP performed spectroscopy analyses. MM and BV performed statistical analysis. SP wrote the first draft of the manuscript. All authors take final responsibility for the decision to submit for publication. All authors had full access to all of the data in the study, took responsibility for the integrity of the data, the accuracy of the data analysis, contributed substantially to the scientific process, leading up to the writing of the paper, and were entirely responsible for the scientific content of the paper.

## Conflict of Interest

The authors declare that the research was conducted in the absence of any commercial or financial relationships that could be construed as a potential conflict of interest.
