# Supplementary material for: Proinflammatory Cytokines Predict Brain Metabolite Concentrations in the Anterior Cingulate Cortex of Patients With Bipolar Disorder
Source: Front Psychiatry. 2020 Dec 8;11:590095. doi: 10.3389/fpsyt.2020.590095 (PMC7753118; doi:10.3389/fpsyt.2020.590095)
Supplement: Supplementary file 1 [file Data_Sheet_1.pdf]

S_Table 1. Coefficients estimates for 5000 bootstrapped elastic net regression predicting GSH concentrations.

	mean ±sd	Lower CI	Higher CI	VIP
Intercept	0,29±0,09	0,12	0,46	100
Age	0,02±0,07	-0,12	0,15	42,24
Sex	0,02±0,04	-0,06	0,09	54,1
Onset	0,14±0,13	-0,11	0,39	80,24
n. of episodes	0,16±0,11	-0,06	0,39	88,46
BMI	0,00±0,06	-0,13	0,12	38,08
Lithium	0,02±0,06	-0,09	0,13	43,8
IL-1	-0,01±0,09	-0,20	0,18	34,7
IL-1ra	0,01±0,10	-0,19	0,20	35,06
IL-2	-0,05±0,15	-0,34	0,25	29,64
IL-4	0,03±0,12	-0,21	0,26	25
IL-5	-0,01±0,16	-0,32	0,30	14,48
IL-6	0,14±0,42	-0,68	0,96	52,14
IL-7	-0,04±0,13	-0,29	0,21	38,94
IL-8	-0,16±0,47	-1,08	0,77	43,9
IL-9	-0,02±0,08	-0,18	0,14	33,16
IL-12-p70	-0,05±0,16	-0,38	0,27	37,36
IL-13	0,31±0,42	-0,51	1,13	65,64
IL-17	0,16±0,17	-0,18	0,50	73,9
IFN-γ	-0,01±0,14	-0,29	0,26	22,54
TNF-α	-0,24±0,20	-0,63	0,16	80,94
CCL2	0,03±0,10	-0,17	0,23	33,62
CCL3	-0,04±0,12	-0,27	0,20	33,06
CCL4	0,16±0,15	-0,13	0,46	77,62
CCL5	-0,04±0,09	-0,22	0,14	37,78
CCL11	-0,11±0,17	-0,43	0,22	52,34
CXCL10	-0,07±0,17	-0,40	0,25	46,18
bFGF	0,00±0,07	-0,13	0,13	30,14
C-CSF	0,11±0,18	-0,24	0,47	52,7
PDGF-bb	0,07±0,11	-0,15	0,29	54,08

S_Table 2. Coefficients estimates for 5000 bootstrapped elastic net regression predicting Myo-Inositol concentrations.

	mean ±sd	Lower CI	Higher CI	VIP
Intercept	0,34±0,13	0,08	0,59	100
Age	0,22±0,13	-0,04	0,49	94,76
Sex	-0,01±0,06	-0,12	0,09	48,08
Onset	0,17±0,17	-0,17	0,51	75,44
n. of episodes	-0,03±0,10	-0,22	0,16	45,56
BMI	0,01±0,08	-0,15	0,17	44,36
Lithium	-0,04±0,07	-0,17	0,10	60,5
IL-1	0,16±0,16	-0,15	0,47	76,64
IL-1ra	0,06±0,13	-0,20	0,32	44,02
IL-2	0,05±0,23	-0,41	0,50	16,84
IL-4	0,32±0,30	-0,27	0,90	85,18
IL-5	0,02±0,14	-0,25	0,29	18,3
IL-6	0,14±0,23	-0,31	0,59	63,96
IL-7	-0,05±0,18	-0,39	0,30	36,84
IL-8	-0,29±0,83	-1,92	1,33	33
IL-9	-0,10±0,15	-0,39	0,20	53,08
IL-12-p70	-0,01±0,16	-0,33	0,31	22
IL-13	-0,08±0,27	-0,62	0,46	32,9
IL-17	0,10±0,16	-0,21	0,42	52,98
IFN-γ	-0,04±0,20	-0,44	0,36	26,08
TNF-α	0,01±0,14	-0,26	0,29	25,16
CCL2	-0,01±0,13	-0,26	0,24	34,06
CCL3	0,02±0,15	-0,27	0,32	31,32
CCL4	0,04±0,12	-0,20	0,28	37,86
CCL5	-0,11±0,14	-0,38	0,17	66,02
CCL11	-0,11±0,20	-0,50	0,29	41
CXCL10	-0,06±0,24	-0,53	0,41	48,7
bFGF	-0,21±0,18	-0,57	0,14	82,16
C-CSF	0,06±0,18	-0,29	0,40	29,94
PDGF-bb	0,02±0,10	-0,18	0,22	27,26

S_Table 3. Coefficients estimates for 5000 bootstrapped elastic net regression predicting NAA concentrations.

	mean ±sd	Lower CI	Higher CI	VIP
Intercept	0,30±0,08	0,15	0,46	100
Age	-0,05±0,10	-0,25	0,16	51,72
Sex	0,01±0,04	-0,06	0,08	41,14
Onset	0,00±0,06	-0,12	0,12	29,02
n. of episodes	-0,01±0,06	-0,13	0,12	42,58
BMI	-0,06±0,10	-0,26	0,14	48,48
Lithium	0,01±0,05	-0,07	0,10	34,52
IL-1	-0,01±0,07	-0,15	0,12	24,46
IL-1ra	-0,02±0,10	-0,22	0,18	33,08
IL-2	-0,13±0,23	-0,58	0,32	55,32
IL-4	0,02±0,12	-0,21	0,25	17,34
IL-5	0,01±0,13	-0,24	0,25	8,74
IL-6	0,13±0,23	-0,32	0,58	58,62
IL-7	0,01±0,12	-0,23	0,25	27,84
IL-8	-0,08±0,45	-0,95	0,80	23,92
IL-9	0,02±0,09	-0,17	0,20	34,68
IL-12-p70	-0,08±0,20	-0,48	0,31	41,34
IL-13	0,10±0,26	-0,40	0,61	33,76
IL-17	-0,01±0,08	-0,17	0,16	24,48
IFN-γ	-0,01±0,15	-0,30	0,28	18,6
TNF-α	-0,07±0,12	-0,30	0,17	47,66
CCL2	-0,02±0,09	-0,20	0,16	30,4
CCL3	0,02±0,11	-0,21	0,24	17,84
CCL4	0,00±0,08	-0,15	0,15	26,8
CCL5	0,15±0,14	-0,12	0,43	77,6
CCL11	-0,02±0,09	-0,20	0,16	22,7
CXCL10	0,03±0,13	-0,22	0,28	30
bFGF	-0,01±0,06	-0,13	0,11	28,68
C-CSF	0,04±0,14	-0,23	0,31	28,94
PDGF-bb	0,01±0,07	-0,13	0,15	27,4
